# High-density rhesus macaque oligonucleotide microarray design using early-stage rhesus genome sequence information and human genome annotations

**DOI:** 10.1186/1471-2164-8-28

**Published:** 2007-01-23

**Authors:** James C Wallace, Marcus J Korth, Bryan Paeper, Sean C Proll, Matthew J Thomas, Charles L Magness, Shawn P Iadonato, Charles Nelson, Michael G Katze

**Affiliations:** 1 University of Washington, Department of Microbiology, Seattle, WA 98195-8070, USA; 2 Washington National Primate Research Center, Seattle, WA 98195-8070, USA; 3 Illumigen Biosciences, Inc., 201 Elliott Ave. West, Suite 500, Seattle, WA 98119, USA; 4 Agilent Technologies, Inc., 395 Page Mill Rd. Palo Alto, CA 94306, USA

## Abstract

**Background:**

Until recently, few genomic reagents specific for non-human primate research have been available. To address this need, we have constructed a macaque-specific high-density oligonucleotide microarray by using highly fragmented low-pass sequence contigs from the rhesus genome project together with the detailed sequence and exon structure of the human genome. Using this method, we designed oligonucleotide probes to over 17,000 distinct rhesus/human gene orthologs and increased by four-fold the number of available genes relative to our first-generation expressed sequence tag (EST)-derived array.

**Results:**

We constructed a database containing 248,000 exon sequences from 23,000 human RefSeq genes and compared each human exon with its best matching sequence in the January 2005 version of the rhesus genome project list of 486,000 DNA contigs. Best matching rhesus exon sequences for each of the 23,000 human genes were then concatenated in the proper order and orientation to produce a rhesus "virtual transcriptome." Microarray probes were designed, one per gene, to the region closest to the 3' untranslated region (UTR) of each rhesus virtual transcript. Each probe was compared to a composite rhesus/human transcript database to test for cross-hybridization potential yielding a final probe set representing 18,296 rhesus/human gene orthologs, including transcript variants, and over 17,000 distinct genes. We hybridized mRNA from rhesus brain and spleen to both the EST- and genome-derived microarrays. Besides four-fold greater gene coverage, the genome-derived array also showed greater mean signal intensities for genes present on both arrays. Genome-derived probes showed 99.4% identity when compared to 4,767 rhesus GenBank sequence tag site (STS) sequences indicating that early stage low-pass versions of complex genomes are of sufficient quality to yield valuable functional genomic information when combined with finished genome information from a closely related species.

**Conclusion:**

The number of different genes represented on microarrays for unfinished genomes can be greatly increased by matching known gene transcript annotations from a closely related species with sequence data from the unfinished genome. Signal intensity on both EST- and genome-derived arrays was highly correlated with probe distance from the 3' UTR, information often missing from ESTs yet present in early-stage genome projects.

## Background

The rhesus macaque (*Macaca mulatta*) serves as a model for many facets of human development and physiology and is one of the most widely used nonhuman primates for the study of infectious diseases, such as AIDS. The widespread use of this species in biomedical research led to a proposal in 2002 to generate its complete genome sequence [1]. This proposal was followed up by low-pass whole-genome shotgun (WGS) sequencing of rhesus in 2004 and the completion of a preliminary draft of the genome in January 2005 [2]. The importance of rhesus and related macaque species as experimental animals has prompted us to use the genome sequence to develop a macaque-specific microarray to provide the requisite tools for global gene expression profiling and functional genomic analyses.

Until recently, we and others have been limited to using human sequence based microarrays for experiments aimed at analyzing gene expression in rhesus and other macaque species [3-10]. Although not optimal, this approach is feasible because of the relatively close evolutionary distance between the two species. Rhesus and human species diverged approximately 25 million years ago, and the nucleotide similarity between rhesus and human is estimated at 95% [11]. Despite this similarity, nucleotide mismatches between the species can confound expression analysis on commonly used microarray platforms. For example, experiments using mixed-species cDNA microarrays required raising expression fold-change thresholds to a level that limited the number of genes whose expression can be confidently measured [12]. Similarly, an Affymetrix GeneChip analysis measuring presence of gene expression by hybridizing rhesus mRNA on human chips required twice the signal intensity for Affymetrix analysis software to indicate gene presence compared with using human mRNA, rendering many human GeneChip probe sets unusable when analyzing rhesus samples [13]. In silico approaches have also been used to try to improve the reliability of using human probe sets to study cross-species gene expression by informatically "masking" probes that show excessive cross-species nucleotide probe mismatching [14,15]. Even single base-pair mismatches occurring on Agilent 60-mer oligonucleotide microarrays may result in as much as a 50% drop in test/reference signal intensity if the mismatches fall in the 5' region of the oligonucleotide probe [16].

**Figure 1:**
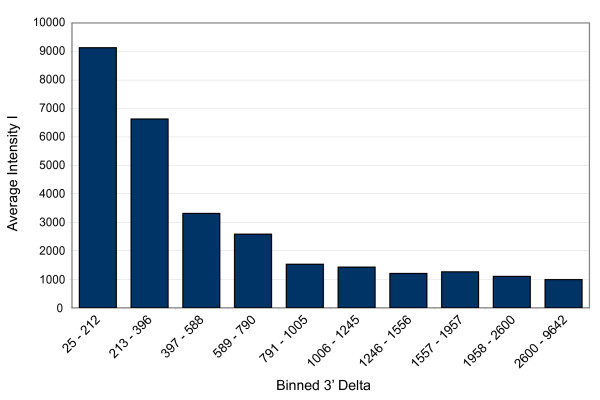
Average binned signal channel intensities for 60-mer oligonucleotide probes increase with proximity to the 3' UTR of gene transcripts.

We previously reported on the use of macaque EST sequences to develop the first commercially available two-color oligonucleotide rhesus-specific microarray, Katze Rhesus Macaque 1 (KRM1) [11]. Over 36,000 EST sequences from eleven rhesus tissues were used for probe design. KRM1 gene coverage represented only 3,500 distinct rhesus/human RefSeq [17] orthologs out of a possible 23,000, largely due to the redundancy that is characteristic of tissue-specific EST libraries as well as difficulties in achieving effective mRNA transcript coverage of unique EST contigs for probe design. To supplement the EST-derived gene list, an additional 500 RefSeq gene probes on the microarray were designed from sequence tag site (STS) sequences generated by leveraging human transcript and genomic information [18], and approximately 1,000 human probes were added to the array from the Agilent Human HA1v2 microarray to include sequences important in virus-host interaction pathways. EST sequence assemblies often did not extend to the 3' untranslated region (UTR) of an orthologous human gene, rendering probe design regions proximal to the 3' edge of genes unavailable. Due to 3' labeling bias, Agilent Technologies recommends designing probes within 800 bp of the 3' end of a gene transcript [Nelson personal communications]. Figure 1 shows the effect of probe distance from the terminal exon 3' UTR for our KRM1 EST-based array. Probe sequences more than 400 bp from the 3' end of the human gene ortholog showed a dramatic attenuation of signal intensity with the best hybridization signals occurring closest to the 3' end of the gene.

For the above reasons, there was clearly a need for an alternative to our EST-based array to provide better coverage of rhesus/human gene orthologs and to optimize probe selection regions to include genomic regions proximal to the 3' end of gene transcripts. In the present study, we employed a human/rhesus comparative genomics approach to address these issues, using human genome sequence and annotation to derive optimal probe design regions from an unfinished and highly fragmented build of the rhesus genome sequence. This approach greatly increased the number of genes available for expression analysis and optimized hybridization signal intensities. These results show that early stage genome projects are a valuable source of information that can be immediately utilized for functional genomics assays.

## Results and discussion

Earlier studies report that the terminal exon of human transcripts, which contains the optimal 3' UTR microarray probe design region, averages almost 1,400 bp with a median length of 1,000 bp [18]. If probe hybridization efficiency is related to the distance of a probe from the 3' UTR, it means that the great majority of all optimal microarray probe design regions fall within the terminal exon of human genes. We tested the conservation of rhesus/human 3' UTRs by first deconstructing the human genome into its constituent exons. We extracted sequence data for each exon from the May 2004 build of the human genome using RefSeq version 11 exon coordinates for 22,975 genes provided by the UCSC genome browser annotation [19]. These 248,000 exon sequences, and their predicted exon order and orientation for each gene, were used to construct a human RefSeq exon sequence database. We found a mean length of 1,233 bp for the terminal exon and 3' UTR of RefSeq genes including single-exon genes and excluding genes with unassigned chromosomal locations. Terminal exon lengths ranged from 14 bp up to a maximum exon length of 12,800 bp with a median length of 916 bp.

We matched human genome transcript information to rhesus unfinished contigs by initially comparing 22,975 terminal exon sequences derived from RefSeq with 486,000 Baylor rhesus genome project version 0.1 WGS contigs averaging 5,540 bp in length, excluding rhesus assemblies containing highly repetitive elements. Remarkably, we were able to match 22,797 out of the 22,975 human terminal exon sequences (99%) to the unfinished rhesus genome sequence, including 17,702 out of 17,934 (99%) distinct rhesus/human RefSeq gene orthologs. Mean alignment size was approximately 1,100 bp covering greater than 90% of each human terminal exon. Human terminal exon sequences matched rhesus contigs with a 94.4% mean identity.

### Rhesus microarray design and probe quality assessment

Microarray design requires comparing all individual probes with all transcriptome sequences to exclude probes highly similar to more than one location in the transcriptome and therefore subject to non-specific mRNA cross-hybridization [20]. To address this, we created a "virtual" rhesus transcriptome by aligning 224,000 non-terminal human RefSeq exons with the same 486,000 rhesus WGS contigs used to determine the probe design regions described above. Again, we were able to align non-terminal exons for over 99% of human RefSeq genes with a mean alignment size of 154 bp and a human/rhesus 96.5% mean identity. Best matching human/rhesus exon sequences were concatenated in order and orientation based on the human transcript information to create a virtual rhesus transcriptome to test probe regions for cross-hybridization potential. To guard against missing pieces of the early stage WGS genome, we created a more conservative composite rhesus/human transcriptome database, which we used to test for cross-hybridization. Probes were designed and arrayed at Agilent Technologies resulting in 18,296 rhesus/human RefSeq ortholog gene probes, including transcript variants, which represent over 17,000 distinct genes. These 18,296 rhesus genome-derived probes were included in the final array layout, with one probe per gene. The new 22,500-probe custom rhesus array, Katze Rhesus Macaque 2 (KRM2), is commercially available from Agilent Technologies [21], Agilent Microarray Design Identification (AMADID) Number 013791. Affymetrix Corporation has also recently developed a commercial rhesus-specific microarray based on the November, 2004 version of the Baylor rhesus genome project along with GenBank rhesus EST, STS and mRNA sequences up to March 30, 2005 [22].

We tested the similarity of the KRM2 genome-derived probes to other available rhesus sequences to gauge the quality of WGS contigs used in probe design. We searched each KRM2 probe against a BLAST database of 4,767 high-quality GenBank STS sequences derived from rhesus PCR products designed specifically to 3' UTR regions [18]. We found that 3,977 KRM2 60-mer probes matched rhesus STS sequences with a mean similarity of 99.4%, demonstrating high sequence quality in 3' UTR probe design regions despite the overall low 3.5× genome coverage (R. A. Gibbs personal communications) in the January 2005 build of the rhesus genome.

### Distance of probes from transcript 3' UTRs

**Figure 2:**
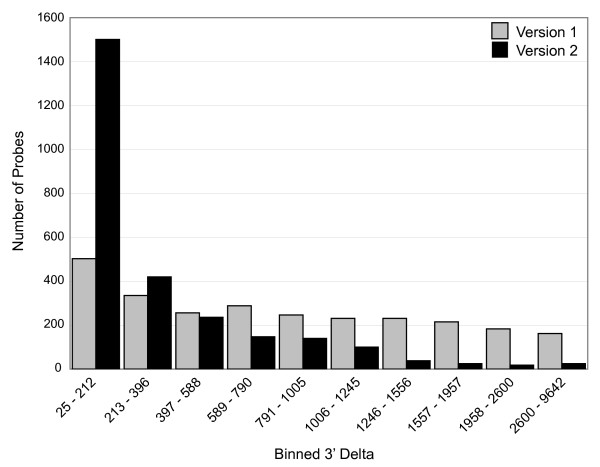
Genome-based rhesus probes on microarray KRM2 are closer to the 3' UTR of gene transcripts compared with rhesus EST-based probes on the KRM1 microarray.

Figure 2 displays the distribution of microarray probes in relation to the distance from the 3' UTR for all transcripts present on both the KRM1 and KRM2 arrays. KRM1 60-mers showed a mean distance of 1,084 bp from the 3' UTR of their rhesus/human transcript ortholog genes when BLAST searched, whereas KRM2 probes averaged less than 400 bp, with half of the probes falling less than 200 bp from the predicted end of the orthologous transcript.

### Comparison of KRM1 and KRM2 rhesus arrays

We tested the KRM2 microarray by hybridizing cRNA probes generated from equal mass amounts of mRNA derived from rhesus brain and spleen (hybridized in two channels with an experimental *n *= 4). The same probes were also used to replicate the experiment on our EST-based KRM1 microarray. Brain and spleen tissues were chosen for the experiment to maximize potential differential expression of KRM1 probes, which were mainly designed using rhesus brain and spleen tissue EST sequences. A minimum two-fold difference in test/reference channel signal intensity and a maximum *P *value of 0.01 were selected as cut offs to indicate differential gene expression for individual probes.

Using these criteria, 1,463 genes showed differential expression on the KRM1 microarray, compared with 4,826 genes showing differential expression on the KRM2 microarray. Although the number of differentially expressed genes will obviously change according to experimental samples and treatments, this helps demonstrate the effect of the four-fold increase in the number of unique genes available on the KRM2 array. In addition, 60-mer probes for 2,650 human RefSeq gene orthologs are common to both the KRM1 and KRM2 arrays, which allowed us to compare cy3/cy5 signal channel and fold-change measurements for each of these probes.

When we compared 2,650 genes present on both the KRM1 and KRM2 arrays, 1,085 genes were differentially expressed on the KRM2 array versus 841 differentially expressed genes on KRM1, a 22% improvement using the new array. Of the 841 genes differentially expressed on KRM1 732 of the identical genes (87%) showed differential expression on the KRM2 array. The greater number of differentially expressed genes on KRM2 versus KRM1 for genes represented on both arrays suggests a distinct improvement in probe performance using genomic versus EST based array probe design. Despite this improvement, overlap in expression results between identical genes on KRM2 and KRM1 is, as expected, not perfect, and microarray results will continue to be used, by our lab and others, as part of a discovery process requiring individual gene verification by methods such as RT-PCR. Hybridization results for all rhesus probes for both arrays in the above experiment are given in supplemental Tables 1 and 2 [see Additional files 1 and 2].

**Figure 3:**
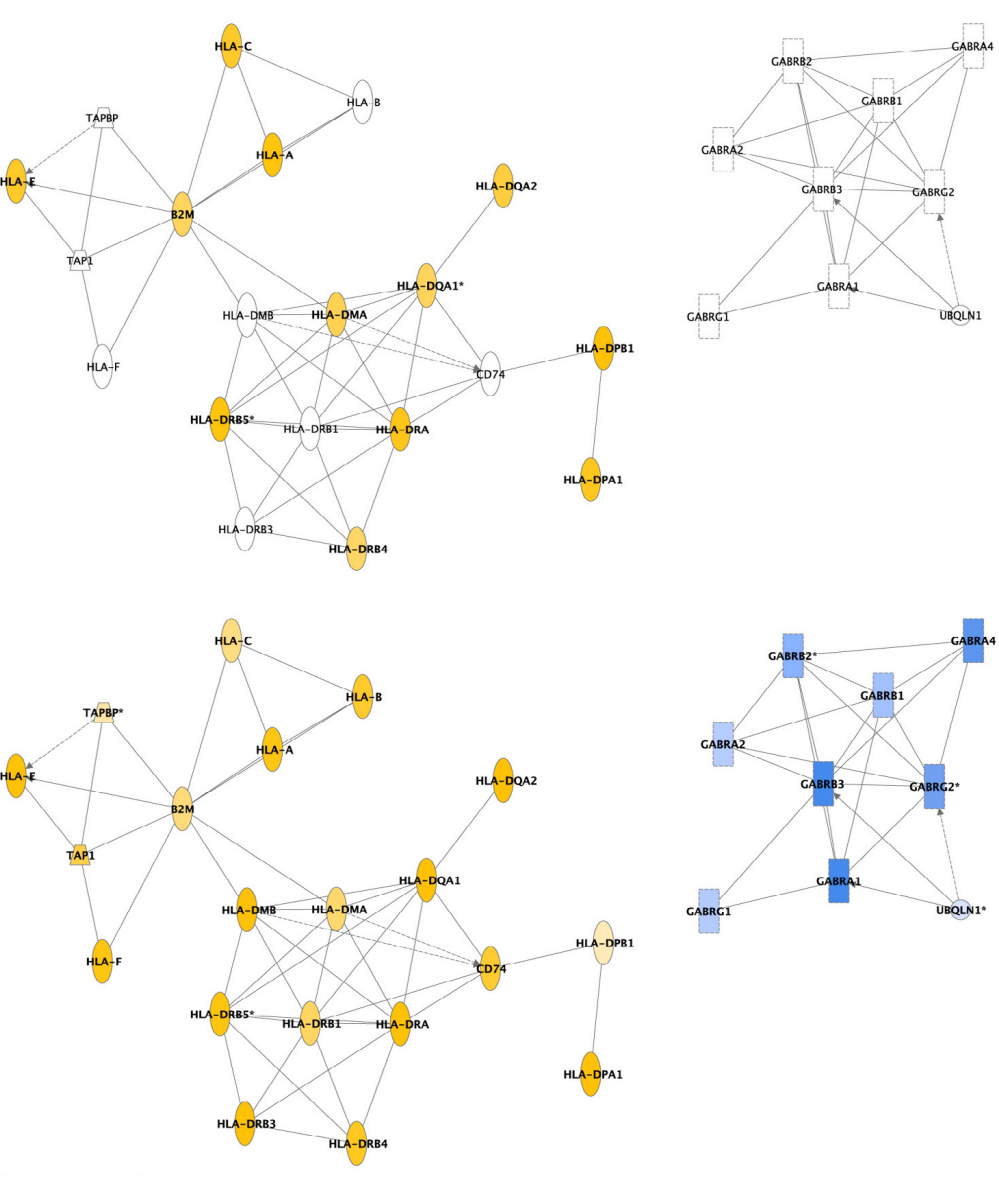
Comparison of gene coverage between KRM1 and KRM2 for GABA receptor signaling (brain-centric) and antigen presentation (spleen-centric) pathways highlights greater coverage and specificity of probes on the KRM2 array designed from genomic data. These networks are laid out with the KRM1 results on top and the KRM2 below. Genes indicated in yellow were more prominently expressed in spleen while the blue denotes higher expression levels in brain. The antigen presentation network (in yellow) shows a 66% greater coverage on the KRM2 array, while the GABA receptor signaling network (in blue) is completely void on KRM1 and fully covered on KRM2.

The results of the brain versus spleen hybridizations highlight the extra coverage garnered in the KRM2 array. Figure 3 shows genes differentially expressed between the two tissue sets in the GABA receptor signaling and antigen presentation pathways, as determined using Ingenuity Pathway Analysis software [23]. As expected, the cluster of GABA receptor signaling genes was more highly expressed in the brain tissue, whereas the cluster corresponding to antigen presentation was expressed at higher levels in spleen. We saw a 40% better representation of genes in the antigen presentation pathway using the KRM2 array, and a 100% increase in representation of the genes in the GABA pathway. This is of particular interest since one of the macaque EST libraries that we sequenced to design the KRM1 array was brain derived. Although our EST sequences did contain data for 5 of the 9 GABA-related genes, sufficient sequence data for probe design was available for only three of these genes (GABRA2, GABRB1, and UBQLN1). It is therefore clear that use of the early stage genome information provides better sequence information for probe design as well as a greater depth of coverage in terms of overall numbers of genes.

## Conclusion

**Figure 4:**
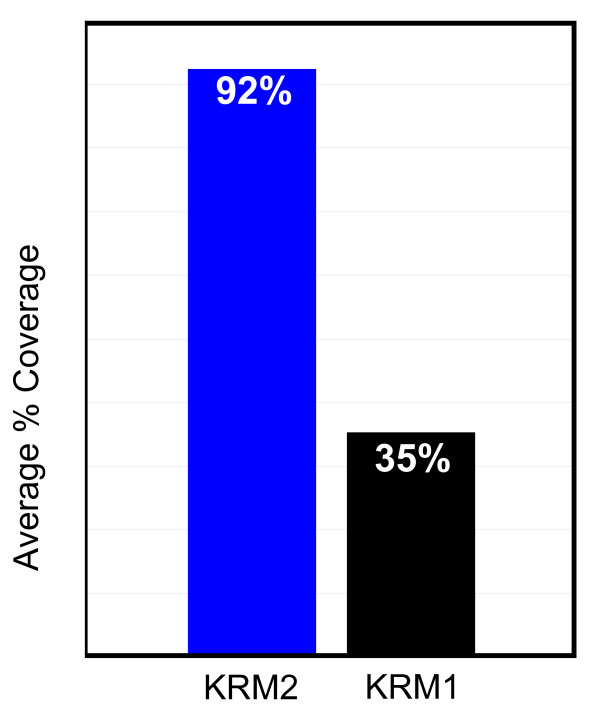
Average coverage per pathway, where at least one gene is present, for the 540 pathway maps represented in GeneGo's MetaCore database.

One of the greatest benefits of utilizing early stage genome information in conjunction with closely related species annotation in oligonucleotide microarray design is the huge increase in the number of different genes available for mRNA abundance measurements compared with relying on EST sequence data alone. This increased number of genes in turn helps provide more comprehensive input for pathway and network analysis of differential gene expression. Figure 4 shows the average coverage per pathway, where at least one gene is present, for 540 pathway maps represented in GeneGo's MetaCore[24] database for genes available on KRM2 and KRM1 arrays.

The relationship between signal intensity and probe distance from the 3' UTR was much more dramatic than expected and a factor to be seriously considered in microarray probe design as well as microarray analysis. The apparent reason for this bias appears to relate to the efficiency of the reverse transcriptase reaction.

Historically, full-length cDNA transcripts have been challenging to achieve, with most first-strand synthesis reactions resulting in pools of transcripts in the 400 bp to 1.5 Kbp range. Premature terminations of the reaction frequently occur due to secondary and tertiary structures in the transcript, and increased product size is often associated with increases in single-base errors in the sequence [25]. Due to the nature of the reverse transcriptase reaction, the 3' region of the transcript is therefore the best target for probe design considering its greater likelihood of being transcribed accurately and completely. This may be particularly significant when designing probes for transcript variants where alternative exons require probes distal to the end of the gene transcript. The distance of the probe from the 3' UTR may also greatly outweigh other factors such as probe/mRNA mismatching, so that mRNA samples from closely related macaque species including *M. nemestrina *and *M. fascicularis*, can also be used for gene expression analysis on the rhesus genome-based KRM2 microarray. 18,296 KRM2 rhesus probes compared with *M. nemestrina *and *M. fascicularis *EST sequences showed a greater than 98% similarity to both species, representing on average a single base mismatch per 60-mer probe.

Although there are many advantages in leveraging annotations from closely related species to design microarray probes from genomic sequences, there are obvious limitations to this method. Gene isoforms, including splice variants, are restricted to those mapped from the related species annotation; true splice variant information still has to be obtained by other methods, such as cDNA sequencing from different tissues from the microarray target species. Species-specific genes are also not addressed using this method. Important innate immunity genes, such as theta-defensins, are only expressed in Old World monkeys such as rhesus macaque [26], whereas the adaptive immune system HLA-C loci present in humans is missing in macaques [27].

In addition to designing rhesus probes using human genome annotations, we will also continue to use species-specific cDNA-derived probes on future macaque microarrays, particularly in light of the growth of the number of macaque ESTs and full-length mRNA transcripts available in public databases. Currently, almost 1,000 EST-derived probes were carried over from KRM1 onto the new KRM2 microarray. Ultimately, microarray probes derived from macaque-specific unannotated assemblies may be the most interesting of all, since they offer the greatest potential for discovering new genes and gene expression pathways [28].

## Methods

### Bioinformatics pipeline

**Figure 5:**
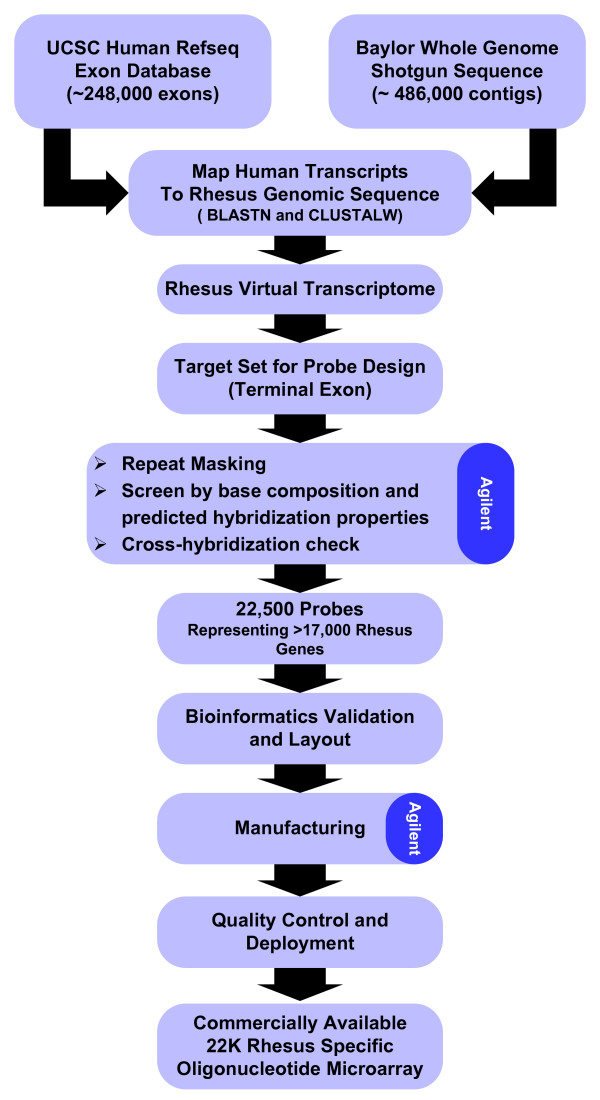
KRM2 rhesus macaque microarray bioinformatics pipeline.

Figure 5 shows bioinformatics components used in array design. Human genome build 34, NCBI RefSeq version 11, and the January 2005 Baylor rhesus build 0.1 genome were used as the reference databases for all analyses. NCBI BLAST for LINUX version 2.2.1 [29] was used for all BLAST searches conducted on four Intel dual-processor computer systems. CLUSTALW [30] version 1.83 was used for secondary alignment of human/rhesus exon high-scoring segment pair regions using default CLUSTALW DNA sequence alignment parameters. BLAST and CLUSTALW parsing, comparisons, and database loading routines were written using Java JDK version 1.4 (Sun Microsystems). MySQL version 4.1 for Microsoft Windows Professional [31] was used as the relational database to store all sequence and analysis information.

A MySQL database table was constructed to store 248,000 NCBI RefSeq exon sequences and exon positions for 23,000 human RefSeq genes. Each human exon sequence was searched using BLASTN against a database of 486,600 Baylor rhesus genome contigs with BLAST parameters of b = 1, v = 1 and expected value of 10^-4^. Human/rhesus High Scoring Pairs (HSPs) for the best matching rhesus contig for each exon were parsed from BLAST output and stored in an exon alignment table. This table was used as input to a Java script to determine the mapping between BLASTN human exon HSPs and the best matching rhesus genome contig for each human exon. The longest matching rhesus HSP was used to establish the initial probe target alignment region between each human exon and the best matching rhesus contig. Additional rhesus HSPs were used to extend the alignment region upstream and downstream from the initial alignment until all HSPs were used. Once a rhesus contig alignment region was established, the DNA sequence for this region was used to produce a full-length alignment with its corresponding human exon DNA sequence using CLUSTALW. Ungapped full-length rhesus genome DNA alignment regions for each gene were then handed off to the probe design process as probe design target sequence candidates. Terminal exons were used whenever possible for probe design due to their proximity to the 3' end of a gene and because they do not cross intron/exon DNA sequence boundaries. A gzipped fasta file of rhesus DNA alignment regions matching terminal exons for 22,797 human RefSeq genes produced by the bioinformatics pipeline using the above process is provided in Additional File 3.

Probes were designed using the commercial probe design process and associated algorithms of Agilent Technologies, Inc. As part of this process, all replicate target sequences were removed along with sequences that were too short for probe design. Target sequences were then vector masked using an Agilent-modified version of the Univec database [32] in conjunction with a BLAST-like algorithm and masking scripts. Target sequences were also repeat masked using RepeatMasker and the primate repeat database obtained from Repbase [33]. A set of candidate probes were selected for each target sequence; choosing those that resided within 800 bp from the sequence's 3' end, in addition to matching an optimal base composition profile. This empirically-determined profile defines an appropriate ratio of nucleic acid sequences (A, T, C, G) to perform optimally on Agilent's *in situ *oligonucleotide microarray platform. Final probes were selected from the candidate set based upon duplex formation stability and cross-hybridization potential with a defined transcriptome database.

A probe design file was created to distribute the probes randomly on an Agilent 22K-featured microarray. The final array design (AMADID# 013791) is available for review and download free of charge for registered users of the Agilent eArray application [34]. A complete list of KRM2 gene names, 60-mer rhesus DNA probe sequences and base pair distances from the 3' UTR of corresponding human RefSeq gene transcripts is included in supplemental Table 3 [see Additional file 4].

### Tissue processing

Spleen and brain tissues were collected at necropsy from healthy adult rhesus macaques and immersed immediately in RNAlater (Ambion Inc.) to preserve the quality of RNA.

Brain tissue was pooled from cerebreum and cerebellum from two macaque females and spleen tissue was pooled from four macaque females and one macaque male.

Tissue samples were then homogenized with Solution D (4 M guanidinium thiocyanate, 25 mM sodium citrate, 0.5% sarcosyl, 0.1 M β-mercaptoethanol). Homogenization with a Kinematica Polytron PT1200 instrument and the model PT-DA1212/2 generator (Kinematica, Switzerland) lasted for 30 seconds in 10 ml round-bottom polypropylene test tubes with 5 ml of solution D. In order to reduce generation of aerosols during this process, the polytron's generator was passed through a hole in the test tube lid that had been drilled in a manner that ensured a tight fit with the instrument. To further minimize possible contact with aerosols, a barrier shield was used in addition to positive air pressure respirators and full protective personal protection equipment. Total RNA was subsequently extracted using RNeasy columns (Qiagen) and the quality and quantity of the total RNA was determined by using a Nanodrop spectrophotometer (Nanodrop Technologies) and the Bionalyzer 2100 system (Agilent Technologies).

### Oligonucleotide microarray hybridization and analysis

Samples of both the spleen and brain tissues were pooled using equal amounts of total RNA. cRNA target production was done with the Agilent Low RNA Input Fluorescent Linear Amplification kit (Agilent Technologies). Slides were scanned with an Agilent DNA microarray scanner, and image analysis was performed using Agilent Feature Extractor Software. Each microarray experiment was done with two technical replicates by reversing dye hybridization for experimental and reference samples. All data were entered into a custom-designed database, Expression Array Manager [35], and then uploaded into Resolver 5.0 (Rosetta Biosoftware), DecisionSite for Functional Genomics (Spotfire, Inc.), and Ingenuity Pathway Analysis (Ingenuity Systems) for analysis and mining. Genes were selected to be included for transcriptional profile based on two criteria: a greater than 99% probability of being differentially expressed (*P *≤ 0.01) and an expression level change of 2 fold or greater. Finally, biological gene sets (referred to as Biosets) were compiled for key cellular processes by selecting genes of interest that were both represented on the microarray and which had Gene Ontology (GO) annotation. In accordance with proposed standards [36], all data described in this report, including sample information, intensity measurements, gene lists, error analysis, microarray content, and slide hybridization conditions, are available in the public domain through Expression Array Manager [35]. Microarray raw data are available at the European Bioinformatics Institute (EBI) ArrayExpress database [37] accession number E-TABM-189.

## Authors' contributions

JCW was responsible for all algorithm, software and database development and computational analyses used in the microarray bioinformatics pipeline up to the final probe-design process. JCW, MJK, and BP were responsible for drafting and editing the manuscript. SCP and MJT provided biological materials for array hybridization and subsequent data analysis. CLM and SPI provided macaque EST sequence data and associated analyses. CN led the final probe-design process for the KRM2 array. MGK conceived of and coordinated the study and gave final approval of the version to be published. All authors read and approved the final manuscript.

## Supplementary Material

Additional File 1 KRM1 hybridization analysis. A Microsoft Excel table showing fold-change and pvalues for brain versus spleen tissues for the KRM1 microarray hybridization results described in this study. Click here for file

Additional File 2 KRM2 hybridization analysis. A Microsoft Excel table showing fold-change and pvalues for brain versus spleen tissues for the KRM2 microarray hybridization results described in this study. Click here for file

Additional File 3 Human/rhesus 3' UTR alignments. A FASTA format file of rhesus DNA sequence alignment regions matching terminal exons for 22,797 human RefSeq genes used as KRM2 probe design candidate regions. Click here for file

Additional File 4 KRM2 probes. A Microsoft Excel table of KRM2 gene names, 60-mer rhesus DNA probe sequences and base pair distances from the 3' UTR of corresponding human RefSeq gene transcripts. Click here for file
